# Integrating postabortion care, menstrual regulation and family planning services in Bangladesh: a pre-post evaluation

**DOI:** 10.1186/s12978-017-0298-1

**Published:** 2017-03-11

**Authors:** Kamal K. Biswas, Erin Pearson, S. M. Shahidullah, Sharmin Sultana, Rezwana Chowdhury, Kathryn L. Andersen

**Affiliations:** 1 Ipas Bangladesh, Eureka Saleha, Flat #A2, Mymenshingh Road, Shahbagh, Dhaka, 1000 Bangladesh; 2 Department of Global Health and Population, Harvard T.H. Chan School of Public Health, 677 Huntington Ave, Boston, MA 02115 USA; 3 Ipas, P.O. Box 9990, Chapel Hill, NC 27515 USA

**Keywords:** Menstrual regulation, Postabortion care, Family planning, Service integration, Bangladesh health system

## Abstract

**Background:**

In Bangladesh, abortion is restricted except to save the life of a woman, but menstrual regulation is allowed to induce menstruation and return to non-pregnancy after a missed period. MR services are typically provided through the Directorate General of Family Planning, while postabortion care services for incomplete abortion are provided by facilities under the Directorate General of Health Services. The bifurcated health system results in reduced quality of care, particularly for postabortion care patients whose procedures are often performed using sub-optimal uterine evacuation technology and typically do not receive postabortion contraceptive services. This study evaluated the success of a pilot project that aimed to integrate menstrual regulation, postabortion care and family planning services across six Directorate General of Health Services and Directorate General of Family Planning facilities by training providers on woman-centered abortion care and adding family planning services at sites offering postabortion care.

**Methods:**

A pre-post evaluation was conducted in the six large intervention facilities. Structured client exit interviews were administered to all uterine evacuation clients presenting in the 2-week data collection period for each facility at baseline (*n* = 105; December 2011–January 2012) and endline (*n* = 107; February–March 2013). Primary outcomes included service integration indicators such as provision of menstrual regulation, postabortion care and family planning services in both facility types, and quality of care indicators such as provision of pain management, provider communication and women’s satisfaction with the services received. Outcomes were compared between baseline and endline for Directorate General of Family Planning and Directorate General of Health Services facilities, and chi-square tests and t-tests were used to test for differences between baseline and endline.

**Results:**

At the end of the project there was an increase in menstrual regulation service provision in Directorate General of Health Services facilities, from none at baseline to 44.1% of uterine evacuation services at endline (*p* < 0.001). The proportion of women accepting a postabortion contraceptive method increased from 14.3% at baseline to 69.2% at endline in Directorate General of Health Services facilities (*p* = 0.006). Provider communication and women’s rating of the care they received increased significantly in both Directorate General of Health Services and Directorate General of Family Planning facilities.

**Conclusions:**

Integration of menstrual regulation, postabortion care and family planning services is feasible in Bangladesh over a relatively short period of time. The intervention’s focus on woman-centered abortion care also improved quality of care. This model can be scaled up through the public health system to ensure women’s access to safe uterine evacuation services across all facility types in Bangladesh.

## Plain english summary

In Bangladesh, induced abortion has historically been provided in family planning facilities, while care for incomplete abortion and miscarriage has been provided in health facilities. The pilot intervention integrated care for miscarriage, induced abortion and postabortion contraception in both health and family planning facilities. Offer of care for miscarriage and induced abortion as well as postabortion contraception resulted in higher quality of care for patients. This study demonstrates that abortion service integration within both health and family planning facilities is possible over a short period of time and can improve service provision for women in Bangladesh.

## Background

Integration of health services improves the health of clients by increasing access to and use of varied services [1, 2]. The World Health Organization (WHO) maintains that a “health system consists of all organizations, people and actions whose primary intent is to promote, restore or maintain health” [3]. In this regard, WHO recommends inter-sectoral action by health staff to strengthen service capacity to improve health outcomes [3]. Abortion care is often excluded from other maternal and reproductive health services such as contraceptive services [1], which results in sub-optimal care for abortion clients who may leave the facility without counseling or offer of family planning methods. Studies evaluating abortion services in developing countries have recommended making family planning services part of routine obstetrics and gynecology services to improve both postabortion and postpartum contraceptive provision, which includes ensuring supply of family planning commodities and that service delivery protocols specify family planning methods be offered at all service sites [4, 5]. There is also evidence that abortion clients are interested in receiving a modern method of contraception after their abortion if offered [6, 7]. A study in Egypt demonstrated that provision of counseling and family planning methods at the abortion facility is more effective in increasing postabortion contraceptive uptake than only providing family planning counseling and referring clients to another site to obtain the method [6].

Despite evidence on the benefits of abortion and family planning service integration, the structure and function of many health systems is not conducive to integration [1]. In Bangladesh, there are two separate directorates under the Ministry of Health and Family Welfare that provide different aspects of abortion care. Menstrual regulation (MR) services, performed within 8–10 weeks of the expected date of menstruation [8], have historically been provided by midlevel providers known as family welfare visitors in Directorate General of Family Planning (DGFP) facilities [9]. Postabortion care (PAC) services, to treat incomplete abortion due to unsafe abortion, incomplete safe abortion or miscarriage, have been provided primarily by physicians in facilities under the Directorate General of Health Services (DGHS) [9]. Both DGHS and DGFP operate facilities at multiple levels of Bangladesh’s health system. Community level healthcare consists of Family Welfare Centers operated by DGFP and Union Health and Family Welfare Centers operated by DGHS [9]. At the sub-district or upazila level, exist the Upazila Health Complexes which receive referrals from the community-level centers, and include a Health Unit operated by DGHS as well as a Family Planning Unit operated by DGFP [9]. Secondary level services are offered at Maternal and Child Welfare Centers under DGFP and District Hospitals under DGHS [9]. Under DGHS, the highest level of services is offered at Medical Colleges which also house NGO Reproductive Health Services Training and Education Program (RHSTEP) clinics providing family planning services. The bifurcated system results in reduced quality of care, particularly for PAC patients. PAC is most often provided by the obstetrician/gynecologists in inpatient wards using dilation and curettage under general anesthesia [10], which is an outdated method of surgical abortion. The World Health Organization (WHO) recommends use of vacuum aspiration or medication abortion utilizing misoprostol-based regimens [11]. These uterine evacuation procedures can be safely performed by midlevel providers [12], which can allow for task shifting of uterine evacuation services from physicians in DGHS facilities to midlevel providers. In addition, PAC patients are significantly less likely to receive postabortion family planning as compared to MR clients, in large part because family planning services are typically not available in DGHS facilities [13]. A study in Jessore district in southwestern Bangladesh found that only 2% of PAC patients, as compared to 55% of MR patients, received postabortion contraception [14].

Abortion is common in Bangladesh, where there is an estimated abortion rate of 37 per 1000 women of reproductive age, compared to an average of only 26 per 1000 in South Asia [15, 16]. The Bangladesh penal code restricts abortion except to save the life of a woman, but since 1979, the Government of Bangladesh allows a procedure known as MR to induce menstruation and establish non-pregnancy [8]. An estimated 653,000 MR procedures were performed in health facilities nationwide in 2010 [15]. An additional 647,000 induced abortions were performed in the same year, the majority of which were unsafe [15], carried out by untrained providers or under unhygienic conditions. It is estimated that 231,400 women suffered from complications following induced abortion in 2010 [15]. Postabortion care (PAC) is provided to women with incomplete abortion due to unsafe abortion, incomplete MR or miscarriage.

Integration of Bangladesh’s bifurcated health system has the potential to improve health outcomes for abortion clients, reducing unwanted pregnancy by increasing access to postabortion family planning services at all health facilities and increasing the quality of uterine evacuation services offered. The government adopted a policy of functional integration of family planning services in all health and family planning sites in 2011 and laid out strategies in 2013 [17, 18]. The 2013 health policy implementation strategies point out the importance of sharing expertise and facilities between DGHS and DGFP for maternal and newborn services to achieve priority Millennium Development Goals [18]. Though uterine evacuation service integration was not the primary focus of these policy changes, they created a scope for integrating missing elements of comprehensive abortion care in facilities under DGHS and DGFP. In response, a pilot project was conducted to demonstrate that integration of MR, PAC and family planning services across DGFP and DGHS facilities was feasible. The present study evaluated the ability of the pilot project to make MR, PAC and family planning services available across DGFP and DGHS facilities, and improve quality of care for women seeking uterine evacuation services.

## Methods

### Project description

The18-month pilot project was designed and implemented with the goal of enhancing collaboration between DGHS and DGFP to integrate MR, PAC and postabortion family planning services at 16 selected facilities. Facilities were selected for the pilot project by DGFP and DGHS representatives in conjunction with program implementers based on existing basic infrastructure for service provision such as a private room in which to perform MR and PAC services and accessibility from main roads to support program implementation. Doctors and midlevel providers, including nurses in DGHS facilities and family welfare visitors in DGFP facilities, were provided with training on WHO-recommended manual vacuum aspiration (MVA) technology for MR and PAC with pain management. Supply of necessary commodities was ensured through existing government channels in DGFP facilities and supplemented by the project in DGHS facilities where family planning commodities were not part of the government supply chain. Overall, the project’s goal was to ensure that providers in each facility, either under the umbrella of DGHS or DGFP, were able to provide MR, PAC and family planning services to any woman who requested the service.

The project objectives for the DGFP system were to: 1) strengthen existing MR services by refreshing skills in MVA and provision of postabortion family planning; 2) introduce quality PAC services using MVA; and 3) support services by family welfare visitors at community-level facilities to provide MR using MVA and strengthen referral to the associated Upazila Health Complex for PAC services. The objectives for the DGHS system were to: 1) introduce MR services at District and Medical College Hospitals; 2) add family planning services to PAC services at District and Medical College Hospitals; 3) introduce quality outpatient PAC services in District and Medical College Hospitals and shift the use of dilation and curettage to MVA and/or medication abortion as recommended by WHO; and 4) introduce MR and PAC service provision by nurses.

Prior to project implementation, baseline assessments were conducted in project sites to understand existing patterns of service provision under the bifurcated health system. This assessment identified opportunities for integrating and upgrading services, including provider training, facility improvements and commodity supply. Next, 30 selected providers participated in training events on clinical skills as well as process changes for integration of services at their facilities. One provider was trained in each of the lower level health facilities, and multiple providers, including physicians and midlevel providers (nurses in DGHS facilities and family welfare visitors in DGFP facilities) were trained in higher level health facilities. After training, a whole site orientation was conducted at each pilot facility to ensure that all staff understood the importance of the issue, proposed changes to service provision, and the process for implementation. Trainers and project staff maintained contact with trained providers over the implementation period by phone and in-person to support quality service provision and address clinical and administrative challenges.

### Data collection

Though 16 facilities were included in the pilot project, data for this evaluation rely on client exit surveys, which were only conducted in the six facilities with at least ten MR or PAC cases per month. Multiple levels of the health system were represented by these six facilities, which included one Medical College Hospital, one District Hospital, two Maternal and Child Welfare Centers and two Upazila Health Complexes. Client exit surveys were not feasible in the ten community-level facilities, known as Family Welfare Centers, which were expected to have less than one MR or PAC case per month. Even though the community-level facilities were not included, the six facilities included in the evaluation were located in both urban and rural areas, including Dhaka city and Narayanganj district in Dhaka division (urban) and Habiganj district in Sylhet division (rural).

Client exit surveys were conducted in-person by trained female data collectors at the six project sites included in the evaluation at two time points. Baseline data were collected at the beginning of the project period (December 2011–January 2012), and endline data were collected during the last 2 months of the project (February–March 2013). Data collectors were posted at the sites during all available clinic hours over a 2-week period, and client exit surveys were administered to all eligible and consenting clients at each facility. Women were eligible to participate if they were at least 18 years of age and had a MR or PAC procedure that day. A total of 105 women participated at baseline, and 107 participated at endline, an average of 15 women per facility.

Client exit surveys are the standard tool used by the implementing NGO in their programs globally to measure quality of abortion services from the woman’s perspective. Client exit surveys assess women’s socio-demographic information, wait time to receive abortion care, procedure information, client-provider interaction and postabortion contraceptive information.

### Data analysis

We first present socio-demographic characteristics, including age, education and marital status in the baseline and endline period. We then present indicators of MR, PAC and family planning service integration by the baseline and endline period in each facility type (DGFP or DGHS) and overall. Service integration indicators include information from the provider on the uterine evacuation service type (MR or PAC), and information from women on the type of provider who treated her, whether postabortion family planning counseling was provided, whether a method was accepted and the type, and the reason for not accepting a method. Finally, we present quality of care indicators, including the provider’s report of the woman’s procedure type (MVA or dilation and curettage) and whether pain management was provided, and women’s report of the time waited to be seen by a provider, satisfaction with the amount of time waited, satisfaction with privacy during treatment, and an overall rating of the care received. A provider communication score was constructed based on 11 yes or no questions about specific aspects of provider communication, such as whether the provider gave information on self-care after the procedure and warning signs of complications. One point was given for each yes response, and the provider communication score ranged from 0 for providers who did not communicate on any of the aspects measured to 11 for providers who communicated on all of the aspects of provider communication assessed. A detailed description of variables used in the analysis can be found in Table 1.

**Table 1:** Indicators used in analysis

Variable	Survey Question (s)	Source
Socio-demographic Characteristics		
Age (Range: 18–42)	How old are you?	Woman’s self report
Education	What is your highest level of education?	Woman’s self report
No education		
Primary		
Secondary or higher		
Marital status	What is your current marital status?	Woman’s self report
Married		
Separated/Divorced		
Widowed		
Unmarried		
MR, PAC and Family Planning Service Integration Indicators		
Uterine Evacuation Service Type	What kind of care did this woman receive at the facility today?	Provider’s report
Menstrual Regulation (MR)		
Postabortion Care (PAC)		
Provider Type	What type of health provider treated you?	Woman’s self report
Doctor (Obstetrician-Gynecologist or Medical Officer)		
Midlevel provider (Family Welfare Visitor or Nurse)		
Counseled on Post-abortion Family Planning	Were you told about family planning during your visit today?	Woman’s self report
Yes		
No		
Post-abortion Family Planning Accepted	Did you receive a family planning method today?	Woman’s self report
Yes		
No		
Type of Family Planning Method Accepted	Which method did you receive?	Woman’s self report
Short-acting method (Condoms, Pills, Injectables)		
Long-acting method (Intrauterine Device, Implant, Sterilization)		
No method		
Reason for Not Accepting a Family Planning Method	Why do you think that you did not receive a method?	Woman’s self report
Did not want a method		
Not offered a method		
Did not have method she wanted		
Will accept a method later		
Have not decided on a method yet		
Other		
Quality of Care Indicators		
Procedure Type	What UE method was used to provide this woman with MR/PAC?	Provider’s report
Manual Vacuum Aspiration (MVA)		
Dilation and Curettage		
Pain Management Provided	Did the woman receive anything for pain?	Provider’s report
Yes		
No		
Time Waited before Being Seen by a Provider	How long did you wait in this facility before you were firstseen by a health care worker today?	Woman’s self report
Less than 1 h		
1–2 h		
3–6 h		
6 or more hours		
Satisfaction with Amount of Time Waited	How do you feel about the amount of time you had to wait for your procedure?	Woman’s self report
Acceptable		
Too long		
Satisfaction with Privacy during Treatment	Were you satisfied with the level of privacy that you had during your treatment at this facility?	Woman’s self report
Satisfied		
Not satisfied		
Provider Communication Score (Range: 0–11)	1. Did the health provider talk to you and explain the different procedure options you had for treating your condition?	Woman’s self report
	2. Did the provider introduce him/herself to you by name?	
	3. Did the provider ask you if you had any questions about the procedure?	
	4. Did the provider give you enough info about your care so that you felt comfortable with the procedure?	
	5. Did you provider tell you how to care for yourself once you get home?	
	6. Did the health provider tell you about the need to avoid sexual intercourse until a few days after bleeding stops?	
	7. Did the provider tell you about warning signs or complications you should look for after leaving the facility that mean you should go to the nearest health center or hospital right away?	
	8. Did the provider tell you that without using a family planning method you could get pregnant again quickly, even before your next menstruation?	
	9. Did the provider say anything to you during the procedure to make you more comfortable?	
	10. Did the health provider assure you that the information that you shared would be kept confidential?	
	11. Were you told about family planning during your visit today?	
Rating of Care Received	Overall, how would you rate the care you received today? Was the care excellent, good, fair or poor?	Woman’s self report
Excellent or good		
Fair or poor		

Less than 10% of values were missing for each variable and means and percentages were calculated among non-missing values. Chi-square tests were used to test for differences between categorical variables, and t-tests were used to test for differences between continuous variables. Significance was assessed at an alpha of 0.05. All analyses were conducted using Stata/SE version 12.

## Results

### Demographic characteristics

Most women participating in this study were age 25 or older; the average age of study participants was 26.6 years at baseline and 27.4 years at endline (Table 2). All participants were married. Education level varied in the sample, with approximately 36% of women with no education, 38% with primary education, and 26% of women with secondary or higher education at both baseline and endline. Characteristics of women interviewed at baseline and at endline were similar with no statistically significant differences observed.

**Table 2:** Demographic characteristics of study respondents at baseline and endline

	Baseline (*n* = 105)		Endline (*n* = 107)		
	*n*	(%)	*n*	(%)	*p*-value
Age					0.270
Less than 25 years	42	(40.0)	35	(32.7)	
25 years or older	63	(60.0)	72	(67.3)	
Age, *mean(SD)*	*26.6*	*(5.7)*	*27.4*	*(5.4)*	0.333
Education Level					0.994
No education	38	(36.2)	39	(36.4)	
Primary	40	(38.1)	40	(37.4)	
Secondary or higher	27	(25.7)	28	(26.2)	
Marital Status					1.000
Married	105	(100)	107	(100)	

### MR, PAC and family planning service integration

During the project period there was a statistically significant increase in women reporting that they received MR services in DGHS facilities, from 0% at baseline to 44.1% at endline (*p* < 0.001) (Table 3). There was also an increase in PAC service provision in DGFP facilities, from 13.5% at baseline to 25.0% at endline, but this increase was not statistically significant (*p* = 0.107). The proportion of uterine evacuation procedures performed by midlevel providers increased during the project period, suggesting that task shifting occurred after the intervention training. In DGFP facilities, the proportion of services by midlevel providers increased from 57.2% at baseline to 100% at endline (*p* < 0.001), and in DGHS facilities, the proportion increased from 17.9% at baseline to 49.1% at endline (*p* = 0.005).

**Table 3:** MR, PAC and family planning service integration indicators at baseline and endline, overall and by facility type

	DGFP facilities					DGHS facilities					Overall				
	Baseline		Endline			Baseline		Endline			Baseline		Endline		
	*n*	(%)	*n*	(%)	*p*-value	*n*	(%)	*n*	(%)	*p*-value	*n*	(%)	*n*	(%)	*p*-value
All Uterine Evacuation Clients	(*n* = 74)		(*n* = 48)			(*n* = 31)		(*n* = 59)			(*n* = 105)		(*n* = 107)		
Uterine Evacuation Service Type					0.107					<0.001					0.656
MR	64	(86.5)	36	(75.0)		0	(0)	26	(44.1)		64	(61.0)	62	(57.9)	
PAC	10	(13.5)	12	(25.0)		31	(100)	33	(55.9)		41	(39.0)	45	(42.1)	
Provider Type					<0.001					0.005					<0.001
Doctor	30	(42.3)	0	(0)		23	(82.1)	30	(50.9)		53	(53.5)	30	(28.6)	
Midlevel provider	41	(57.2)	46	(100)		5	(17.9)	29	(49.1)		46	(46.5)	75	(71.4)	
Counseled on Post-abortion Family Planning					0.419					<0.001					0.830
Yes	73	(98.7)	48	(100)		7	(22.6)	39	(66.1)		80	(76.2)	87	(81.3)	
No	1	(1.3)	0	(0)		24	(77.4)	20	(33.9)		25	(23.8)	20	(18.7)	
Counseled on Post-abortion Family Planning	(*n* = 73)		(*n* = 48)			(*n* = 7)		(*n* = 39)			(*n* = 80)		(*n* = 87)		
Accepted Post-abortion Family Planning					0.075					0.006					0.029
Yes	45	(61.6)	37	(77.1)		1	(14.3)	27	(69.2)		46	(57.5)	64	(73.6)	
No	28	(38.4)	11	(22.9)		6	(85.7)	12	(30.8)		34	(42.5)	23	(26.4)	
Type of Family Planning Method Accepted					0.039					0.010					0.122
Short-acting method	36	(49.3)	23	(47.9)		1	(14.3)	26	(66.7)		37	(46.2)	49	(56.3)	
Long-acting method	9	(12.3)	14	(29.2)		0	(0)	0	(0)		9	(11.3)	14	(16.1)	
No method	28	(38.4)	11	(22.9)		6	(85.7)	13	(33.3)		34	(42.5)	24	(27.6)	
Post-abortion Family Planning Not Accepted	(*n* = 28)		(*n* = 11)			(*n* = 6)		(*n* = 13)			(*n* = 34)		(*n* = 24)		
Reason for Not Accepting a Method					0.036					0.051					0.009
Did not want a method	2	(7.1)	0	(0)		3	(50.0)	1	(8.3)		5	(14.7)	1	(4.4)	
Not offered a method	1	(3.6)	1	(9.1)		0	(0)	2	(16.7)		1	(2.9)	3	(13.0)	
Did not have method she wanted	9	(32.1)	0	(0)		2	(33.3)	0	(0)		11	(32.4)	0	(0)	
Will accept a method later	8	(28.6)	9	(81.8)		1	(16.7)	4	(33.3)		9	(26.5)	13	(56.5)	
Have not decided on a method yet	5	(17.9)	0	(0)		0	(0)	2	(16.7)		5	(14.7)	2	(8.7)	
Other	3	(10.7)	1	(9.9)		0	(0)	3	(25.0)		3	(8.8)	4	(17.4)	

Postabortion contraceptive counseling and method provision also improved during the project period, particularly in DGHS facilities. Two thirds of women reported receiving postabortion family planning counseling at endline in DGHS facilities, compared to only 22.6% at baseline (*p* < 0.001) (Table 3). The proportion of women accepting a contraceptive method increased significantly from 14.3% of women counseled at baseline to 69.2% at endline in DGHS facilities (*p* = 0.006). The proportion also increased in DGFP facilities, but this increase was not statistically significant. In DGFP facilities, a significantly larger proportion of women received long-acting contraceptive methods during the project period. At baseline, only 12.3% of women counseled accepted a long-acting contraceptive method, compared to 29.2% at endline (*p* = 0.039). In DGHS facilities, all women received short- acting methods. Women who did not accept a family planning method were asked the reason. At baseline in DGFP facilities, most women did not accept a method because the facility did not have the method she wanted (32.1%), and in DGHS facilities, the most common reasons for non-acceptance at baseline were that she did not want a method (50.0%) and that the facility did not have the method she wanted (33.3%). At endline, no women in DGFP or DGHS facilities reported that the facility did not have the method they wanted. The most common reason for non-acceptance at endline was that she wanted to wait until later before accepting a method (81.8% in DGFP facilities and 33.3% in DGHS facilities).

### Quality of care

Provider training offered through the intervention focused on improving both clinical and patient care skills, and this study showed improvements in both areas, especially in DGHS facilities. Prior to this project, most uterine evacuation procedures in DGHS facilities were performed using dilation and curettage (87.1%), but after implementation of this project, there was a statistically significant decrease in the proportion of procedures performed using this method (33.9%; *p* < 0.001) (Table 4). In DGFP facilities, the proportion of women receiving pain management increased from 82.4% at baseline to 95.8% at endline (*p* = 0.028). Significant differences in pain management were not observed for DGHS facilities as many procedures were already being provided under general anesthesia. Time spent waiting to receive uterine evacuation services was approximately the same between baseline and endline, but women in DGHS facilities reported higher satisfaction with the waiting time at endline (74.6%) compared to baseline (48.4%; *p* = 0.013). In both DGFP and DGHS facilities at endline, a higher proportion of clients reported being satisfied with the level of privacy during treatment but this difference was not statistically significant. The mean provider communication score increased significantly in both DGFP and DGHS facilities, indicating that provider counseling was more comprehensive at endline. In DGFP facilities, the mean provider communication score increased significantly from 7.4 at baseline to 8.9 at endline (*p* = 0.001). In DGHS facilities, the mean provider communication score also increased significantly from 2.1 at baseline to 5.0 at endline (*p* < 0.001). In DGFP facilities, there was a statistically significant increase in the proportion of women rating the care they received as good or excellent from 74.3% at baseline to 89.6% at endline (*p* = 0.038). In DGHS facilities, the proportion of women rating the care they received as good or excellent also increased significantly from 58.1% at baseline to 79.7% at endline (*p* = 0.030).

**Table 4:** Quality of care indicators at baseline and endline, overall and by facility type

	DGFP facilities					DGHS facilities					Overall				
	Baseline (*n* = 74)		Endline (*n* = 48)			Baseline (*n* = 31)		Endline (*n* = 59)			Baseline (*n* = 105)		Endline (*n* = 107)		
	*n*	(%)	*n*	(%)	*p*-value	*n*	(%)	*n*	(%)	*p*-value	*n*	(%)	*n*	(%)	*p*-value
Procedure Type					0.251					<0.001					0.123
MVA	72	(97.3)	48	(100)		4	(12.9)	39	(66.1)		76	(72.4)	87	(81.3)	
Dilation and curettage	2	(2.7)	0	(0)		27	(87.1)	20	(33.9)		29	(27.6)	20	(18.7)	
Any Pain Management Provided	61	(82.4)	46	(95.8)	0.028	29	(93.5)	58	(98.3)	0.232	90	(85.7)	104	(97.2)	0.003
Time Waited before Being Seen by a Provider					0.147					0.127					0.120
Less than 1 h	44	(59.5)	36	(75.0)		24	(77.4)	44	(74.6)		68	(64.8)	80	(74.8)	
1–3 h	28	(37.8)	12	(25.0)		4	(12.9)	14	(23.7)		32	(30.5)	26	(24.3)	
More than 3 h	2	(2.7)	0	(0)		3	(9.7)	1	(1.7)		5	(4.7)	1	(0.9)	
Satisfaction with Amount of Time Waited					0.506					0.013					0.218
Acceptable	63	(85.1)	43	(89.6)		15	(48.4)	44	(74.6)		78	(74.3)	87	(81.3)	
Too long	11	(14.9)	5	(10.4)		16	(51.6)	15	(25.4)		27	(25.7)	20	(18.7)	
Satisfaction with Privacy during Treatment					0.390					0.212					0.595
Satisfied	66	(89.2)	45	(93.8)		12	(38.7)	31	(52.5)		78	(74.3)	76	(71.0)	
Not satisfied	8	(10.8)	3	(6.2)		19	(61.3)	28	(47.5)		27	(25.7)	31	(29.0)	
Provider Communication Score, *mean(SD)*	*7.4*	*(2.6)*	*8.9*	*(2.3)*	0.001	*2.1*	*(3.0)*	*5.0*	*(3.3)*	<0.001	*5.8*	*(3.6)*	*6.7*	*(3.5)*	0.058
Rating of Care Received					0.038					0.030					0.012
Excellent or good	55	(74.3)	43	(89.6)		18	(58.1)	47	(79.7)		73	(69.5)	90	(84.1)	
Fair or poor	19	(25.7)	5	(10.4)		13	(41.9)	12	(20.3)		32	(30.5)	17	(15.9)	

## Discussion

This evaluation demonstrated that the 18-month pilot project was successful in integrating MR, PAC and family planning services across DGFP and DGHS facilities. MR and PAC services became more evenly distributed in both DGFP and DGHS facilities between baseline and endline. Baseline postabortion family planning counseling and method provision was high in DGFP facilities, and these indicators of integration increased significantly in DGHS facilities. However, provision of postabortion family planning counseling and methods needs continued support, especially in DGHS facilities as a third of women were not counseled at endline and all women accepting a method received a short-acting method. Long-acting postabortion contraceptive methods continued to be underutilized in both DGHS and DGFP facilities at endline. Quality of care improved over the intervention period, including clinical aspects of quality such as increased use of WHO-approved uterine evacuation methods and provision of pain management, and non-clinical aspects such as improvements in provider communication. Findings demonstrate that it is possible to integrate abortion and family planning services over a relatively short intervention period in a bifurcated health system such as Bangladesh’s, and that integration of services through an intervention focusing on woman-centered abortion care results in higher quality of care.

Service integration was successful, but results varied somewhat by facility type. The changes in service integration were particularly salient in DGHS facilities, which were only providing PAC services prior to the project. After integration, approximately one half of clients received MR services, and there was a five-fold increase in postabortion family planning provision. DGFP facilities also improved between baseline and endline, but the effects of integration were more modest as these facilities were already providing postabortion contraceptive counseling and methods to most clients at baseline. The largest improvements in service provision in DGFP facilities were in provision of long-acting postabortion contraceptive methods, but these methods were still likely underutilized as a recent study in Bangladesh found that 57% of MR clients do not want to have another child [19]. In both DGHS and DGFP facilities, there was a decrease in stock-outs of family planning methods by endline with no women reporting that they did not accept a method because the method they wanted was unavailable. The project was successful in ensuring that participating facilities had contraceptive commodities, and to increase sustainability, DGHS should procure family planning commodities directly.

Task shifting from doctors to midlevel providers was observed in both DGFP and DGHS facilities at endline. In DGFP facilities, all uterine evacuation services were provided by midlelvels at endline, and in DGHS facilities approximately half were provided by midlevels. Task shifting in abortion care is recommended as trained midlevel providers are able to provide a similar quality of care, and are more likely to be the primary cadre of providers available at the community level [12]. Task shifting benefits abortion clients and the health system by increasing access to services in rural areas, and by freeing physicians to perform more complex procedures in large urban health facilities [20].

Quality of care also improved in both DGFP and DGHS facilities after the intervention on some indicators. In DGHS facilities, the largest improvement was in the switch from use of dilation and curettage to use of MVA, which is expected to result in fewer complications related to the uterine evacuation procedure [11, 21]. In DGFP facilities, provision of pain management increased significantly between baseline and endline. The training providers received as a part of the intervention focused on woman-centered abortion care that stressed the importance of providing pain management as well as two-way communication and counseling on the abortion procedure, after-care and family planning. As a result, provider communication as well as women’s overall rating of the care they received improved significantly between baseline and endline in both DGFP and DGHS facilities. Though provider communication improved in DGHS facilities, the provider communication score was still low at endline (5.0 in DGHS facilities, compared to 8.9 in DGFP facilities), suggesting that more work is needed to improve counseling for women seen in DGHS facilities.

### Study limitations

This study focused on data collected from client exit surveys. While it is important to capture women’s perspectives on the services they received, the study is limited by self-reported data. Future studies should include observation of MR, PAC and family planning counseling services and include a rating of the provider by a trainer or another provider. Data on post-procedure complications was not collected, and there is a lack of information regarding clinical outcomes such as continued bleeding, infection or retained products of conception. Client exit surveys are also potentially limited by social desirability bias, but the study made an effort to minimize this by hiring trained female data collectors to administer the surveys. This study is also limited by the sample, which is not representative of all MR and PAC clients in Bangladesh. Data were collected from facilities in both urban and rural areas and at multiple levels of the health system, but facilities participating in the pilot project were selected by DGFP and DGHS in conjunction with program implementers and are not representative of the country.

## Conclusions

The pilot project demonstrated that it is possible to successfully integrate MR, PAC and family planning services in Bangladesh over a short period of time to ensure that women are able to access these services at both DGHS and DGFP facilities. Scale-up of integration of uterine evacuation and family planning services is recommended throughout the health system, which would require provider training on woman-centered abortion care as well as MR, PAC and family planning commodity supply in both DGHS and DGFP facilities. Particular attention should be paid to training on counseling in DGHS facilities where after intervention implementation, provider communication was poor and postabortion family planning counseling was still not universal. In addition, long-acting methods were underutilized in DGFP and DGHS facilities, and additional training on long-acting family planning methods should be considered.

## Abbreviations

DGFP: Directorate General of Family Planning
DGHS: Directorate General of Health Services
MR: Menstrual regulation
MVA: Manual vacuum aspiration
NGO: Nongovernmental Organization
PAC: Postabortion care
RHSTEP: Reproductive Health Services Training and Education Program
WHO: World Health Organization
